# Structure of a LOV protein in apo-state and implications for construction of LOV-based optical tools

**DOI:** 10.1038/srep42971

**Published:** 2017-02-17

**Authors:** Vladimir Arinkin, Joachim Granzin, Katrin Röllen, Ulrich Krauss, Karl-Erich Jaeger, Dieter Willbold, Renu Batra-Safferling

**Affiliations:** 1 Institute of Complex Systems, ICS-6: Structural Biochemistry, Forschungszentrum Jülich, 52425 Jülich, Germany; 2 Institut für Molekulare Enzymtechnologie, Heinrich-Heine-Universität Düsseldorf, Forschungszentrum Jülich, D-52426 Jülich, Germany; 3 Institut für Bio- und Geowissenschaften, IBG-1: Biotechnologie, Forschungszentrum Jülich, D-52426, Jülich, Germany; 4 Institut für Physikalische Biologie, Heinrich-Heine-Universität Düsseldorf, D-40225, Düsseldorf, Germany

## Abstract

Unique features of Light-Oxygen-Voltage (LOV) proteins like relatively small size (~12–19 kDa), inherent modularity, highly-tunable photocycle and oxygen-independent fluorescence have lately been exploited for the generation of optical tools. Structures of LOV domains reported so far contain a flavin chromophore per protein molecule. Here we report two new findings on the short LOV protein W619_1-LOV from *Pseudomonas putida*. First, the apo-state crystal structure of W619_1-LOV at 2.5 Å resolution reveals conformational rearrangements in the secondary structure elements lining the chromophore pocket including elongation of the Fα helix, shortening of the Eα-Fα loop and partial unfolding of the Eα helix. Second, the apo W619_1-LOV protein binds both natural and structurally modified flavin chromophores. Remarkably different photophysical and photochemical properties of W619_1-LOV bound to 7-methyl-8-chloro-riboflavin (8-Cl-RF) and lumichrome imply application of these variants as novel optical tools as they offer advantages such as no adduct state formation, and a broader choice of wavelengths for *in vitro* studies.

Light-oxygen-voltage (LOV) flavoproteins belong to the Per-ARNT-Sim (PAS) domain superfamily sharing the canonical PAS protein fold^1^,^2^. Widely distributed in multiple kingdoms of life^3^,^4^,^5^, LOV proteins control a number of cellular responses like phototropism, chloroplast movement, stomatal opening, regulation of circadian rhythms, photo-induced growth patterns and pigment synthesis^6^,^7^,^8^. They function specifically as photosensory modules, typically binding a blue light absorbing flavin chromophore (FMN, flavin mononucleotide; FAD, flavin adenine dinucleotide or RF, riboflavin)^9^,^10^,^11^. The photocycle of LOV proteins is reversible. Light absorption results in the formation of a metastable covalent bond between the C4a atom of the flavin ring and the sulfur of the neighboring cysteine residue^12^,^13^,^14^,^15^. The resulting FMN-cysteinyl adduct decays spontaneously in a time period that varies from seconds to several hours in different LOV proteins^7^,^16^,^17^,^18^,^19^,^20^.

Structures of LOV domains reported in the literature so far contain one flavin chromophore per protein molecule, which forms an FMN-cysteinyl adduct in the light state and is noncovalently bound in the dark state. In most cases, structural data on the light state is obtained from crystals grown in dark that were subsequently exposed to light (here termed photoexcited state), which allows FMN-cysteinyl adduct formation in the crystal, thus providing important information on the light-dependent conformational changes in the vicinity of the chromophore^7^,^20^,^21^,^22^,^23^. Due to the crystal packing constraints, however, this approach limits large conformational changes in tertiary and quaternary structure such as reported for the fully-adapted light state crystal structures of VVD from *Neurospora crassa* and Aureochrome 1a (PtAu1a) from *Phaeodactylum tricornutum* where the crystals were grown under continuous light^24^,^25^. Both of these proteins show light-dependent dimerization. In contrast, structures derived from dark-grown crystals do not explain clearly how chromophore-mediated light absorption leads to allosteric changes in protein conformation that activates the signaling processes^26^. For the rational design and implementation of LOV proteins as optical tools, characterization of all relevant structural states as well as detailed knowledge of events leading to allosteric signaling between sensor and effector domains is required^27^,^28^,^29^. We recently reported crystal structures of both dark and fully light-adapted states of the FMN containing PpSB1-LOV protein, illustrating light-induced conformational changes that support a rotary switch signaling mechanism^16^,^26^.

Both PpSB1-LOV and the W619_1-LOV protein reported herein belong to the family of short LOV proteins from *Pseudomonadaceae*^19^, which share 89% sequence identity (Fig. 1a) and similar structural organization, consisting of a LOV core domain and two non-canonical structural elements, an N-terminal helix A′α and a C-terminal helix Jα. The adduct state lifetimes (τrec) of the two proteins PpSB1-LOV and W619_1-LOV at 20 °C are 40 h^30^ and 7.7 h^current study^, respectively. Molecular determinants of the dark recovery kinetics in LOV proteins are poorly understood; we thus reasoned that a comparative structural analysis of W619_1-LOV and the previously reported PpSB1-LOV structures will provide insights about the differences in dark recovery rates amongst this set of homologous proteins originating from a common ancestor. Surprisingly, the W619_1-LOV protein crystallized in the apo-state. In the current study, we report the first apo-state crystal structure of the short LOV protein W619_1 from *Pseudomonas putida*. High sequence identity with previously reported short LOV proteins such as PpSB1-LOV allows a direct comparison of the individual amino acids lining the chromophore binding pocket in both proteins. The structural integrity of apo-W619_1-LOV in the crystal encouraged us to investigate its binding properties to different flavin chromophores in solution. We demonstrate that the W619_1-LOV apoprotein binds both natural (FMN, FAD, RF) and structurally modified (8-Cl-RF, 7-methyl-8-chloro-riboflavin; 7-Br-RF, 7-bromo-8-methyl-riboflavin; LC, lumichrome) flavin chromophores. The remarkably different photophysical and photochemical properties of W619_1-LOV when bound to 8-Cl-RF or LC offer a new perspective for the design of tailored optical tools.

Among the wider PAS superfamily to which LOV proteins belong, several proteins are reported to possess a promiscuous ligand binding site accepting a variety of different naturally occurring and artificial ligands^31^,^32^. Hereby, the bona fide PAS domain of the hypoxia inducible HIF2α transcription factor (HIF2α PAS-B) was crystallized in the apo-state^33^. Similarities and differences of the apo-state LOV domain structure presented here and the apo structure of HIF2α PAS-B provide additional insights into ligand binding in the PAS superfamily.

## Results

### Crystal structure of the short LOV protein W619_1-LOV in apo-state

**Figure 1:**
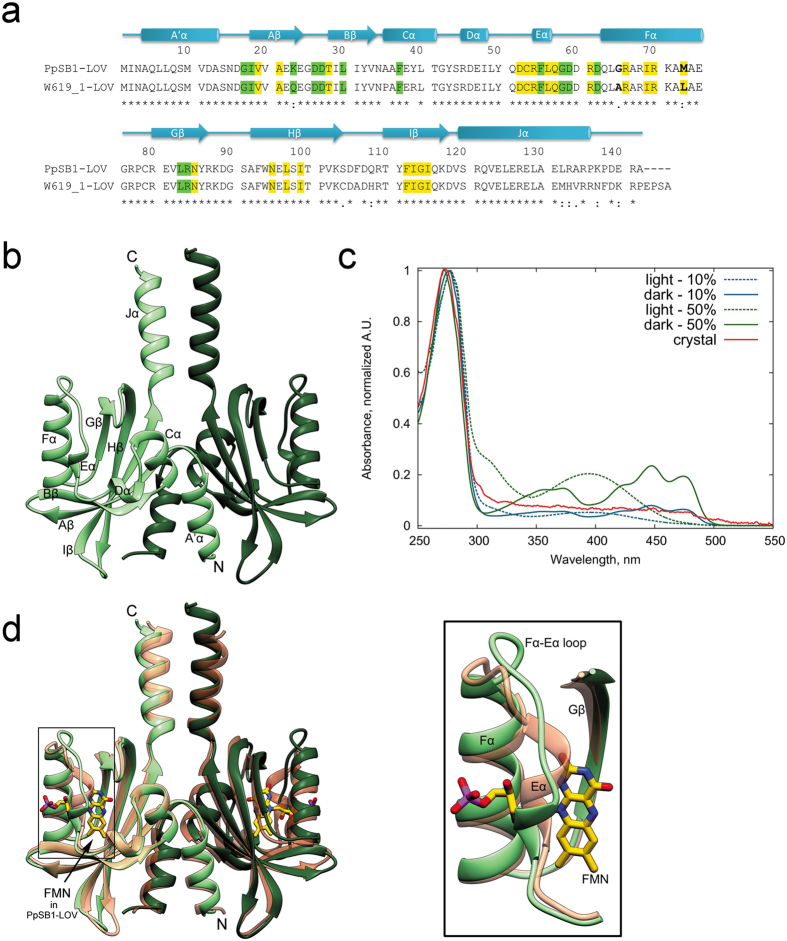
Apo-state crystal structure of W619_1-LOV. (**a**) Sequence and secondary structure alignment of W619_1-LOV and PpSB1-LOV. Secondary structural elements (W619_1-LOV) and residue numbers are indicated above the sequence, where the beta strands from Aβ to Iβ are shown as arrows, and the helices from A’α to Jα are shown as cylinders. The asterisks at the bottom line of the alignment indicate identical residues at a given sequence position, while single and double dots refer to highly and moderately conserved residues, respectively. Residues lining the chromophore pocket at cut-off distances <4 Å and ≥4 Å from FMN in the PpSB1 crystal structure (PDB ID 5J3W) are highlighted in yellow and green, respectively. In the Fα helix, residues showing differences in sequence at position 65 and 73 are indicated in bold. (**b**) Ribbon representation of the W619_1-LOV dimer present in the asymmetric unit. Chains A and B are colored in light and dark shades. (**c**) Absorbance spectra of W619_1-LOV. Single crystal microspectrometry UV/Vis spectrum of a W619_1-LOV crystal shown as solid line in red color does not show any absorbance peaks that are typical for protein-bound flavin chromophores. In contrast, spectra of 10% (blue) and 50% (green) loaded protein solution show the typical absorbance maxima of 390 nm and 447 nm in light (dashed) and dark (solid lines) state, respectively. Spectra are normalized at 280 nm. (**d**) Superposition of W619_1-LOV (green) and PpSB1-LOV dark state (salmon, PDB ID 5J3W) crystal structures. The inset shows conformational differences in the secondary structure elements lining the chromophore pocket such as elongation of Fα helix, shortening of the Eα-Fα loop and partial unfolding of the Eα helix. The chromophore FMN bound to PpSB1-LOV is depicted as stick model and is colored by element: carbon, yellow; nitrogen, blue; oxygen, red; phosphorus, purple. Chains A and B are colored in light and dark shades.

W619_1-LOV was overexpressed in *E. coli* BL21 (DE3) and purified as described previously^19^. The chromophore loading of the purified protein was determined to be ~10%, which could be improved up to ~50% *in vitro* by incubating the purified protein solution with ten-fold excess of FMN (see Methods). HPLC analysis reveals that the recombinant protein contains 96% FMN and 4% RF (Fig. S1). Crystallization setups were performed with both 10% and 50% FMN-loaded protein, where both protein preparations yielded tetragonal crystals within a week that were colorless, indicating absence of chromophore. The final structure contains two molecules forming a dimer per asymmetric unit (Fig. 1b, Table S1). In the corresponding electron density maps, no density could be assigned to the flavin chromophores FMN or RF. The absence of chromophore in the crystal was also verified by single crystal microspectrometry measurements on W619_1-LOV crystals, which showed no characteristic absorption in the wavelength range between 300 and 500 nm (Fig. 1c). In contrast, typical dark and light state spectra were obtained for the protein in solution irrespective of chromophore loading.

The crystal structure of W619_1-LOV revealed an overall fold similar to the previously published homolog PpSB1-LOV in the dark state (Fig. 1d)^26^. Electron density is well-defined for the residues 1–134 (1–137 in Chain B) with additional residues of the purification tag at the N-terminus in Chain B, which are numbered −9 to 0 for consistency in numbering of LOV domain residues in published data. Each monomer comprises a LOV core domain (aa 17–118) that shows a typical α/β PAS fold, and two helical elements at the N- (aa 1–13) and the C-terminus (aa 120–133 in chain A/120–136 in chain B) (Fig. 1b). Interestingly, the W619_1-LOV dimer interface is similar to that observed in the dark state structure of PpSB1-LOV (Fig. 1d). Based on PISA analysis^34^, the buried surface area of 3272 Å^2^ in W619_1-LOV is comparable to 3096 Å^2^ in the dimer of PpSB1-LOV.

**Figure 2:**
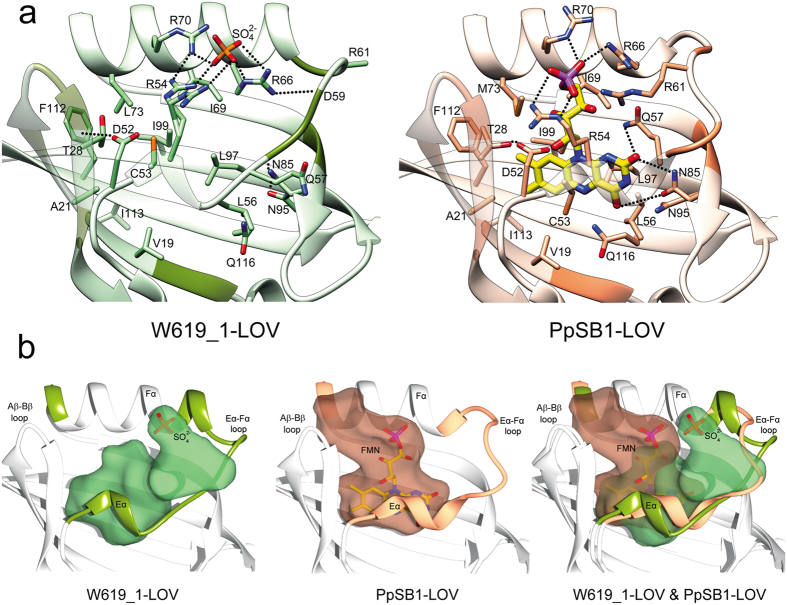
Comparison of the chromophore binding pocket of W619_1-LOV and PpSB1-LOV. (**a**) Ribbon representations of W619_1-LOV (*left panel*, transparent green) and PpSB1-LOV (*right panel*, transparent salmon). The FMN-interacting protein residues lining the chromophore pocket within 4 Å cut-off distance are shown as stick models and >4 Å cut-off distance are shown as dark colored ribbon segments in PpSB1-LOV and for comparison, in the apo-state of W619_1-LOV. The sulfate ion in W619_1-LOV is shown as a stick model colored by element - oxygen, red; sulfate, orange. FMN bound to PpSB1-LOV is shown as stick model colored by element as in Fig. 1d. (**b**) Solvent accessible cavities created with 1.4 Å probe radius represented as transparent surface in W619_1-LOV (*left panel*, green) and PpSB1-LOV (*middle panel*, salmon). Sulfate ion and FMN molecule were not included for cavities calculations but are shown to indicate their positions in the respective structures. Superposition of W619_1-LOV and PpSB1-LOV (*right panel*) showing alignment of the two cavities, which differ in the two structures: extending towards the Aβ-Bβ loop and C-terminal end of the Fα helix in PpSB1-LOV, and towards the Eα-Fα loop and the N-terminus of the Fα helix in W619_1-LOV.

The apo-state W619_1-LOV structure displays significant conformational differences compared to its FMN-containing homolog PpSB1-LOV around the chromophore pocket as illustrated by the superposition of the two structures (Fig. 1d). Most significant changes are seen in the Eα helix, and the Eα-Fα loop region (residues 52–65), with root-mean-square distance (rmsd) values of 2.65 Å for the Cα atoms of the respective Eα-Fα loops. Notably, the Eα helix (53–55) contains the conserved photoactive cysteine C53, that is highly relevant for adduct formation (Fig. 1d). This 3_10_ helix is partially unfolded in the W619_1-LOV structure, shortening it by two residues (aa 53–55 compared to aa 53–57 in PpSB1-LOV). The photoactive C53 shows a different rotamer conformation, with the Sγ atom facing away from the chromophore pocket (Fig. 2a). The succeeding Fα helix is extended by two residues starting at position 62 compared to 65 in PpSB1-LOV according to DSSP secondary structure assignment^35^, resulting in shortening of the Eα-Fα loop (Fig. 1d). Conformational changes are illustrated in the Supplementary Movie morphing between the apo-state from the current study, and the FMN-bound conformation based on the PpSB1-LOV structure (Supplementary Movie M1).

A comparison of residues lining the chromophore binding pocket in crystal structures of W619_1-LOV and PpSB1-LOV is shown in Fig. 2a. In the W619_1-LOV apo-state, the residues involved in hydrogen bond interactions with the chromophore in PpSB1-LOV point away from the pocket or form intramolecular interactions, as seen for the key residues Q116, N95 and N85. Likewise, the residues L56, Q57 and R61 located on the Eα-Fα loop, which usually interact with the ribityl chain and the phosphate group in FMN containing LOV proteins point away from the pocket. In contrast, positions of hydrophobic residues (V19, A21, I69, L97, I99, F112, I113 and G114) in the chromophore pocket remain unchanged. Additionally, other residues (Q23, D26, D27 and T28) of the Aβ-Bβ loop show side chain rotamer changes, where the latter shows hydrogen bond interactions to D52 that precedes the Eα helix.

Notably, the FMN molecule of PpSB1-LOV when superposed onto the binding site of W619_1-LOV no longer fits due to steric clashes and the above-mentioned conformational changes in the residues lining the chromophore binding pocket. While no flavin-specific electron density could be detected in W619_1-LOV, two buffer molecules - a sulfate and a chloride ion fit well in the traces of density detected in the vicinity in chain A and chain B, respectively (Fig. 2a). Even though different in position (*right panel*, Fig. 2b), the ions form salt bridge interactions with arginine residues R54, R66 and R70, resembling the interaction between the FMN phosphate group and residues of the arginine cluster seen in the FMN containing PpSB1-LOV structures^16^,^26^. In the PpSB1-LOV structure, four arginine residues form a unique network of hydrogen bond interactions with the FMN phosphate group *right panel/*, (Fig. 2a). This close network of hydrogen bonds was proposed to stabilize the FMN molecule bound in the chromophore pocket, affecting the dark recovery kinetics indirectly^16^. The authors conclusion was based on the mutagenesis experiments where two of these arginines (R61 and R66) when mutated in PpSB1-LOV resulted in acceleration of the dark recovery by a factor of 280. The four arginine residues are conserved in the PpSB1 and W619_1 LOV sequences (Fig. 1a). With the exception of R61 located in the Eα-Fα loop, which is significantly different in conformation, the Cα atoms of the other three arginine residues are well-superimposable in the two crystal structures. It is intriguing that the two proteins show significant differences in chromophore loading (~10% in W619_1-LOV and > 80% in PpSB1-LOV), indicating that additional factors other than the arginine cluster play a critical role in determining the binding properties of the chromophore in the two proteins.

We next determined the solvent accessible area for W619_1-LOV and PpSB1-LOV by probing the binding pocket with a standard 1.4 Å radius^36^ as described in the methods section. The obtained three-dimensional envelope resembles the van der Waals shape of an FMN isoalloxazine ring system and most of its ribityl chain (Fig. 2b). In PpSB1-LOV, the pocket extends towards theAβ-Bβ loop and the C-terminal end of the Fα helix. In contrast, in W619_1-LOV it extends towards the Eα-Fα loop and the N-terminus of Fα helix. These differences are likely related to the conformational changes seen in the apo-state. First, the position of the FMN phosphate group (as in PpSB1-LOV) is taken up by protein residues R54 and R70 in W619_1-LOV, which causes closure of the pocket. Second, the aforementioned elongation of the Fα helix at the N-terminal end and shift in the Eα-Fα loop create more cavity space in the vicinity (top right in the right panel of Fig. 2b).

Crystallization of the apo-state in the presented work is favored by the crystallization buffer conditions. It is conceivable that the conformational changes seen in Eα, Fα helices, and the Eα-Fα loop are a consequence of the absence of chromophore. The sequence alignment of W619_1-LOV and PpSB1-LOV shows differences beyond position 132 in the C-terminus of the Jα helix, and a small set of changes (eight aa residues in total) in the LOV core domain (Fig. 1a). The C-terminal residues are likely unstructured as they could not be traced in the electron density maps of both crystal structures^current study^^26^. Additionally, spatial separation of the Jα helix residues from the chromophore binding site in the core domain indicates that the C-terminal residues are unlikely to play a decisive role in chromophore binding. For the LOV core domain, no structural changes were observed in the crystal structures that could be correlated to the differences in aa sequences, with the exception of A65 and L73 on the Fα helix (G65 and M73 in PpSB1-LOV). Both positions are highly divergent in LOV proteins. In the apo-state, L73 is well superimposable on its equivalent M73 in PpSB1-LOV, where the latter is involved in hydrophobic interactions with the FMN molecule (Fig. 2a). Located in the N-terminal half of the Fα helix, A65 forms an additional H-bond with D62 (A65-*N*…*O*−62D, 2.97 Å), causing elongation and stabilization of the Fα helix at its N-terminal end. This could trigger conformational changes seen in the preceding Eα-Fα loop and Eα helix. Alanine has been previously shown to stabilize the helix structure relative to glycine by 0.4 to 2 kcal mol^−1 ^37. Site-directed mutagenesis experiments combined with structural and spectroscopic characterization of the respective mutants are, however, required to verify the role of individual residues in stabilization of the apo-state, as seen in W619_1-LOV protein crystals.

### Binding of different flavin derivatives to the W619_1-LOV apo-protein

**Figure 3:**
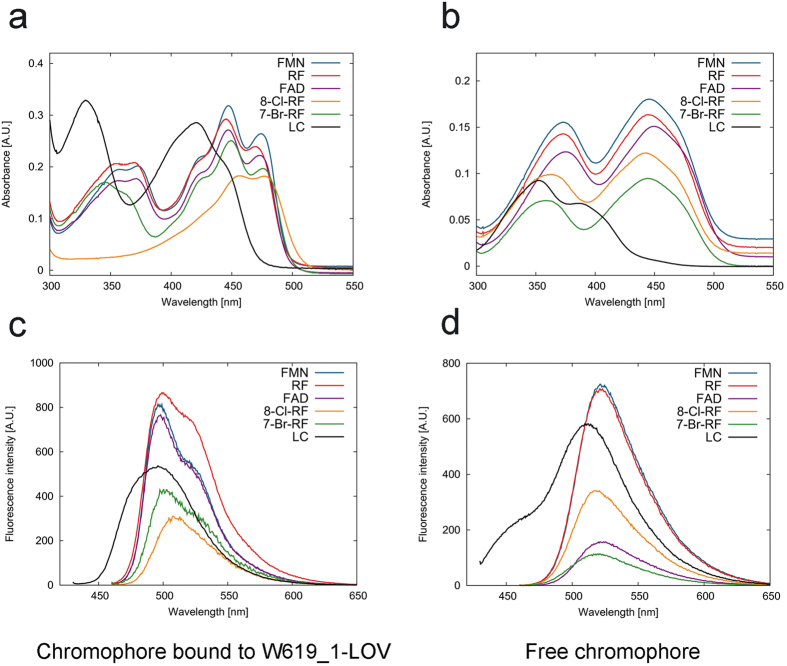
Absorption and fluorescence spectra of the W619_1-LOV protein reconstituted with flavin chromophores. (**a**) Absorption spectra of W619_1-LOV bound to natural (FMN, RF, FAD) and structurally modified flavin chromophores (8-Cl-RF, 7-Br-RF, LC) (**b**) Absorption spectra of free chromophores used in panel a. (**c**) Fluorescence emission spectra of W619_1-LOV bound to natural (FMN, RF, FAD) and structurally modified flavin analogs (8-Cl-RF, 7-Br-RF, LC). (**d**) Fluorescence emission spectra of free chromophores used in panel **c**. Fluorescence emission measurements were done by excitation at a wavelength of 450 nm for all samples, except for LC where it was 420 nm.

In the last decade, LOV proteins have been used extensively for the design of optogenetic tools and LOV-based fluorescent reporters^27^,^28^,^29^,^38^,^39^,^40^. One of the desirable properties for the construction of LOV-based optical tools is the tuning of photophysical properties such as absorption and fluorescence emission maximum in LOV-based fluorescent proteins. The apo-state crystal structure presented in the current study reveals that the missing chromophore does not affect the structural integrity of the W619_1-LOV protein. We therefore developed a protocol for the production of chromophore-free W619_1-LOV. The total chromophore release was based on denaturation using guanidinium chloride as described in the methods section. Refolded protein was loaded with the desired flavin chromophore, and the resulting chromophore-bound proteins were characterized for their absorption, fluorescence and photocycle properties. The chromophores tested in the current study were FMN, RF, FAD, 8-Cl-RF, 7-Br-RF and LC. While FMN, FAD and RF are typically bound in LOV proteins as natural chromophores, 8-Cl-RF and 7-Br-RF are the artificial flavin derivatives, and LC exists as a photodegradation product of riboflavin (Fig. S2). As shown in Table 1 and Fig. 3, all chromophores bind to the W619_1-LOV apo protein. W619_1-LOV bound to FMN, RF, FAD and 7-Br-RF possessed a functional photocycle exemplified by a dark state absorption maximum around 450 nm (Fig. 3), bleaching upon illumination, and fully reversible dark recovery. These results are consistent with previously reported reconstitution studies performed using the YtvA LOV protein with some of the flavin chromophores (FMN, RF, FAD) common in both studies^41^. In contrast, absorption spectra of the W619_1-LOV bound to 8-Cl-RF and LC showed a shift of ~10 nm and ≥ 25 nm compared to the free chromophores, respectively (Fig. 3a, b). Excitation at 450 nm yielded fluorescence emission spectra with emission maxima at ~495 nm, which is typical for LOV proteins. While we do not observe adduct formation for W619_1-LOV loaded with LC and 8-Cl-RF, we clearly detect flavin specific fluorescence under steady state conditions (Table 1 and Fig. 3c). Moreover, the fluorescence quantum yield of the protein-bound flavin was in all cases as high as that of the respective free chromophore (Table 1). This suggests that the 8-Cl-RF and LC loaded proteins do not show a canonical LOV photocycle but instead directly decay upon excitation from the excited singlet (S1) state to the ground state, which is accompanied by the emission of light in form of fluorescence. Although unlikely, we presently cannot rule out that adduct formation and adduct decay occur on a fast ns or μs time scale for 8-Cl-RF and LC. While most flavin-derivatives bound to W619_1-LOV showed a Stokes shift of ~50 nm, LC bound to W619_1-LOV showed a ~75 nm difference (λ_ex_ ≈ 420 nm) between excitation and emission maximum values (Table 1). Changes in the spectral properties such as these are remarkable and could provide several advantages in the application of LOV proteins as novel optical tools.

## Discussion

For the rational design of optical tools, a detailed knowledge of the protein structure as well as investigations of photochemical and photophysical properties are required. High-resolution structure information on several LOV proteins is available where one flavin chromophore is present per protein molecule. To the best of our knowledge, the apo-state crystal structure of W619_1-LOV reported here is the first apo-state structure of a LOV protein. Although the molecular details of the flavin-protein interactions in various LOV proteins have previously been reported in different structural states such as dark, light, photoexcited, and fully-light adapted, the apo-state structure presented here provides new insights into the nature of flavin recognition. In general, two strategies have been applied for alteration of the optical properties of LOV domains for their application as fluorescence reporters: site-directed mutagenesis, and replacement of the native chromophore with structurally modified flavins. The W619_1-LOV apoprotein binds both natural (FMN, FAD, RF) and modified (8-Cl-RF, 7-Br-RF, LC) flavin chromophores, with remarkably different photophysical and photochemical properties when bound to 8-Cl-RF or LC. In this context, the results presented here offer a new perspective for the design of tailored optical tools. Previously, FMN-bound LOV proteins have been employed as fluorescent reporters where the photoactive cysteine is usually replaced with either an alanine or a glycine, thus abolishing the canonical LOV photocycle^38^,^39^,^40^. Because no adduct formation can take place in the corresponding LOV variants, de-excitation preferentially proceeds by relaxation from the excited singlet S1 state to the ground state of FMN, which is accompanied by the emission of light in form of fluorescence. While the photoactive cysteine (C53 in *Pseudomonas putida* W619_1 and PpSB1; C62 in *Bacillus subtilis* Ytva, C108 in *Neurospora crassa* VVD) is indispensable for adduct formation and has long been assumed essential for the LOV domain signaling response, recent work of Yee and coworkers showed that LOV photoreceptors devoid of the photoactive cysteine can still elicit a functional response both *in vitro* and *in vivo*^42^. The authors demonstrated that flavin photoreduction, which results in the formation of the neutral semiquinone radical and hence protonation of the flavin N5 atom, is sufficient to trigger a LOV domain signaling response, albeit with reduced magnitude compared to wildtype cysteine-containing LOV photoreceptors. These recent observations stress the need for variants of the LOV domain that are inactivated by means other than substitution of the photoactive cysteine, such as by using LOV proteins containing structurally-modified flavins that apparently do not undergo adduct formation (for example, 8-Cl-RF and LC), or by introducing substitutions that impair chromophore binding while maintaining the overall LOV domain structural integrity. However, toxicological properties of these compounds remain unexplored and will need to be investigated for their compatibility with *in vivo* experiments.

Another advantage of the ‘inactive’ variants W619_1-LOV…8-Cl-RF, and W619_1-LOV…LC is that they offer a broader choice of absorbance wavelength that has so far been limited to the absorbance maximum of naturally occurring flavoproteins (~450 nm). Combination of a set of different LOV protein-chromophore complexes could hereby provide opportunities for multicolor fluorescence applications or could enable more complicated ‘*in vitro*’ experiments using. e.g., Förster resonance energy transfer (FRET)-based optical tools. In addition, the W619_1-LOV protein bound to LC shows a fluorescence quantum yield value (Φ_F_) of 0.4 (Table 1), which is significantly high amongst LOV-based FMN-binding fluorescent proteins (FbFPs) with the highest reported values of 0.44 and 0.51 for iLOV and CreiLOV proteins, respectively^27^,^38^,^40^,^43^. Still, these values are lower than the reported Φ_F_ of ~0.6 for green fluorescent protein (GFP). Despite the LOV-specific advantages over GFP-derived fluorescence proteins, further improvements are required regarding their photophysical properties such as absorption and fluorescence emission maxima, fluorescence quantum yield, photosensitization, and photostability.

LOV domains are a subset of the PAS domain superfamily^2^,^44^. The canonical structure of the LOV core domain consists of ~110 amino acids forming a typical α/β PAS fold composed of a central five stranded antiparallel β-sheet and several α-helices, flanking the sheet. In nature, PAS domains are known to bind a variety of small molecule ligands such as heme, flavins (FMN and FAD), 4-hydroxycinnamic acid (4-HCA), divalent metal cations, C3-C4 carboxylic acids (malonate, malate, succinate), C6 carboxylic acids (citrate)^31^. Hereby, the bound ligand determines the signaling response of the respective sensory domain ranging from sensing metabolites (carboxylic acids), oxygen (heme), redox potential (FAD) and physical signals such as light (FMN, FAD and 4-HCA)^31^. In addition, members of PAS family such as the PAS domain of the aryl hydrocarbon receptor (AhR) possess a promiscuous ligand binding site accepting a variety of different naturally occurring and artificial ligands^32^. The C-terminal PAS-B domain of the hypoxia inducible HIF2α transcription factor has been crystallized in an apo form without a bound ligand^33^. While the solution NMR structure of the same protein in the apo-state revealed a well-defined PAS core structure, portions of the Fα helix and the subsequent Fα-Gβ loop were poorly defined in the NMR structure ensemble^45^. Moreover, NMR relaxation experiments suggested that the Fα helix, Fα-Gβ loop, and Hβ strand are inherently flexible as evidenced by high R2 and low ^15^N (^1^H) NOE values^45^. While the natural ligand remains elusive, screening approaches identified a number of artificial ligands that can bind to the HIF2α PAS-B domain^33^, suggesting that the ligand binding site is promiscuous accepting a variety of ligands. In HIF2α PAS-B this site is comprised of Eα and Fα helices and the Gβ, Hβ, and Iβ strands of the central PAS β scaffold forming a 290 Å^3^ cavity which contains eight ordered water molecules stabilized by a hydrogen-bonding network with buried polar residues of the protein. In contrast, in the W619_1-LOV apo structure no ordered water molecules are observed, the hydrophobic side chains (on Hβ and Iβ) that interact with the dimethyl benzene part of the flavin isoalloxazine ring in PpSB1-LOV largely retain their conformation whereas the polar side chains are reoriented, thereby stabilizing the cavity that might be filled by unordered water molecules not observed in the crystal structure. In particular, C53 (Eα), Q116 (Iβ), Q57 (Eα-Fα loop in W619_1-LOV, at the end of Eα in PpSB1-LOV) and R66 (Fα) occupy a different position in the W619_1-LOV apo structure compared to the recently published dark state PpSB1-LOV structure (Fig. 2a). The structural rearrangement is most pronounced for Q57, which in PpSB1-LOV is in hydrogen bonding distance to the FMN-O2 atom and the FMN-ribityl O4 atom. In the apo-state, Q57 of W619_1-LOV flips completely out of the pocket (Fig. 2a). In conclusion, similar structural regions involving residues on Eα, Fα, Hβ and Iβ are involved in ligand recognition, which is not surprising given the structural conservation within the PAS family of proteins. The results presented in this study provide a basis for improvement in designing LOV-based optogenetic tools, which considering the similarities and structural conservation between LOV and PAS domains, might be extendable to the members of PAS superfamily.

## Methods

### Protein expression and purification

Expression of a W619_1-LOV coding cDNA (Swiss Prot: B1J385) was conducted as described previously^7^,^19^. Briefly, the N-terminally His-tagged gene (tag sequence: MGSSHHHHHHSSGLVPRGSH) was expressed in *Escherichia coli* BL21(DE3) in autoinduction media prepared with Terrific-Broth-Medium (X972, Carl-Roth, Karlsruhe, Germany) supplemented with 50 μg/ml kanamycin. For induction, 0.5 g/L glucose and 2 g/L lactose were added. Typically, overexpression was carried out in 250 mL cultures for 72 h at 15 °C with constant agitation (110 rpm). For higher chromophore loading, the media was supplemented with 50 μM riboflavin (A6279, AppliChem GmbH, Darmstadt, Germany) and the temperature was increased to 37 °C for 3 h, after which the cultures were shifted to 30 °C for 21 h. Protein purification was performed as described previously^7^. Pure fractions in storage buffer (10 mM Tris-HCl pH 8.0, 10 mM NaCl) were pooled, supplied with 3 mM Tris(2-carboxyethyl)phosphin (TCEP) and concentrated using Vivaspin concentrator units (molecular mass cutoff: 10 kDa) (Sigma-Aldrich, St. Louis, MO, USA). In order to achieve higher chromophore loading *in vitro*, 1 mM of protein sample was mixed with FMN (F6750, Sigma-Aldrich, St. Louis, Missouri, USA) to a final concentration of 10 mM and the mixture was incubated overnight in the dark at 4 °C. Unbound chromophore was removed by size-exclusion chromatography (SEC) using a HiLoad 26/600 Superdex 200 pg column (GE Healthcare, Buckinghamshire, UK) on an ÄKTA pure FPLC system at room temperature. Eluted fractions were pooled, supplemented with 3 mM TCEP and concentrated to the desired protein concentration as mentioned above.

### Protein crystallization

Concentrated protein (~32 mg/ml) was used for the crystallization setups using the vapor-diffusion method. Crystals were grown in dark conditions in 1.8 μl sitting drops (0.9 μl purified protein + 0.9 μl reservoir solution) against 70 μL of 100 mM MES pH 6.0–6.3 and 1 M ammonium sulfate. Typically, colorless protein crystals appeared within a week at 14 °C.

### Data collection and structure determination

Single crystals were cryoprotected by stepwise addition of 35% (v/v) glycerol, mounted in a loop under dim red light and flash cooled with gaseous nitrogen at 100 K. X-ray diffraction data at 100 K was recorded at the beamline ID29 (ESRF, Grenoble, France^46^) tuned to a wavelength of 0.9763 Å on a PILATUS 6 M detector (Dectris Ltd., Baden, Switzerland). Even though both 10% and 50% FMN-loaded protein resulted in well-diffracting crystals, the crystal selected for data collection was from 10% FMN-loaded protein as it diffracted to a higher resolution. Data collection strategy was based on calculations using the program BEST which accounts for radiation damage and symmetry^47^. Data processing was conducted using the program XDS^48^. The space group of W619_1-LOV crystals was determined to be I4_1_ with POINTLESS (part of CCP4 package^49^). Analysis of the Matthews coefficient suggested two molecules per asymmetric unit, with a Matthews coefficient of 3.76 Å^3^/Da and a solvent content of 67%. The initial phases were obtained by molecular replacement using MOLREP (part of CCP4 package), where the search model was created from the dark state crystal structure of PpSB1-LOV (PDB ID: 5J3W) by replacing amino acid side chains. The model was further improved with several cycles of refinement using the PHENIX package^50^ and manual rebuilding with the program COOT^51^. Protein geometry analysis revealed no Ramachandran outliers, with 100% residues in favored regions. The overall MolProbity score was 1.76 (within the 100th percentile for overall geometric quality among protein crystal structures of comparable resolution)^52^. Data collection and refinement statistics are listed in Table S1.

### Single crystal microspectrometry

W619_1-LOV crystals grown in the dark were mounted in a loop under dim red light and flash cooled with gaseous nitrogen at 100 K. An absorbance spectrum in the wavelength range 250–650 nm was recorded using an UV/Vis microspectrometer at ID29S (ESRF, Grenoble, France^53^).

### Apo protein production

In order to obtain the apo form, dark adapted W619_1-LOV protein sample was denatured by dilution in 6 M guanidine hydrochloride, 20 mM NaH_2_PO_4_/Na_2_HPO_4_ pH 6.5 and washed a few times by subsequent dilution and concentration steps in Vivaspin concentrator units (molecular mass cutoff: 3 kDa) to remove the chromophores thoroughly. Refolding was done by rapid dilution with the storage buffer in two steps - first to ~1 M guanidine hydrochloride with 5 min incubation at room temperature, followed by dilution to ~0.1 M guanidine hydrochloride. The refolded protein was concentrated and the buffer was exchanged to 10 mM NaH_2_PO_4_/Na_2_HPO_4_ pH 8.0, 10 mM NaCl for spectroscopic studies. Reconstitution of FMN and all other chromophores used in this study was performed as described above in the section ‘Protein expression and purification’. Chromophores LC, 8-Cl-RF, 7-Br-RF and FAD were purchased from Sigma-Aldrich, St. Louis, Missouri, USA.

### Determination of chromophore loading

Chromophore loading was measured as described previously^54^, with the exception of the presence of 6 M guanidine hydrochloride (in buffer 20 mM NaH_2_PO_4_/Na_2_HPO_4_ pH 6.5) that was mixed with protein in a ratio of 5:1. Due to denaturation of the protein with simultaneous release of the chromophore, we could do the following calculations. First, the concentration of the chromophore was derived from the absorption value at 447 nm and the FMN molar attenuation coefficient ε_FMN447nm_ of 11 825 M^−1^ cm^−1^ (determined by authors). Second, the absorption contribution of the chromophore at 280 nm was subtracted from the total value by using the FMN molar attenuation coefficient ε_FMN280 nm_ of 20 670 M^−1 ^cm^−1^ (determined by authors). By using the corrected absorption at 280 nm, the molar concentration of the protein was determined using the molar attenuation coefficients for amino acids in 6 M guanidine hydrochloride, 20 mM NaH_2_PO_4_/Na_2_HPO_4_ pH 6.5 (Trp = 5 690 M^−1^ cm^−1^, Tyr = 1 280 M^−1^ cm^−1^, Cys = 120 M^−1^ cm^−1^)^55^. Chromophore loading was expressed as ratio between protein and chromophore concentrations in percent.

### Spectroscopic techniques

Spectroscopic measurements were carried out with a Shimadzu UV-1800 UV-Vis spectrometer (Shimadzu, Kyoto, Japan). For generation of the light state, the protein (in buffer 10 mM NaH_2_PO_4_/Na_2_HPO_4_, 10 mM NaCl pH 8.0) was illuminated for at least 30 s using a 450 nm blue-light LED with a radiant power of 50 mW (Luxeon Lumileds, Phillips, Aachen, Germany). Dark recovery kinetics was measured from the illuminated sample by recording the absorption recovery at 475 nm, as described previously^30^.

### Measurement of solvent-accessible cavities

Cavities were extracted as void volume from two protein surfaces where “external surface” is created by a rolling sphere of 10 Å radius, whereas a probe radius of 1.4 Å was used for the “internal surface” using the 3 V web server^36^. All ligand/ion molecules were removed from the structure before performing the calculations.

### Graphical representation

Unless otherwise stated, figures and morphing movie were generated with UCSF Chimera^54^ using secondary structure assignments given by the DSSP program^35^. UV-Vis and fluorescence spectra were plotted with Gnuplot^56^.

## Additional Information

**Accession codes:** Atomic coordinates and structure factors for W619_1-LOV have been deposited in the Protein Data Bank (http://www.rcsb.org/pdb/) under PDB ID 5LUV.

**How to cite this article:** Arinkin, V. *et al*. Structure of a LOV protein in apo-state and implications for construction of LOV-based optical tools. *Sci. Rep.* **7**, 42971; doi: 10.1038/srep42971 (2017).

**Publisher's note:** Springer Nature remains neutral with regard to jurisdictional claims in published maps and institutional affiliations.

## Supplementary Material

Supplementary Information

Supplementary Movie

## Figures and Tables

**Table 1:** Photophysical and photochemical properties of W619_1-LOV bound to flavin chromophores.

Chromophore	Binding	Photocycle	Dark recovery^a^ τ (min) at 20 °C	Absorbance λ_max_(nm)		Fluorescence^b^λ_max_ (nm)		Quantum yield^b^ Φ_*F*_	
				bound	free	bound	free	bound	free
FMN	yes	yes	460 ± 10	447	445	496	524	0.28	0.27
RF	yes	yes	1.5 ± 0.1	445	445	500	524	0.29	0.27
FAD	yes	yes	54.0 ± 0.5	447	450	499	523	0.25	0.03
7-Br-RF	yes	yes	28.5 ± 0.5	449	444	502	520	0.03	0.04
8-Cl-RF	yes	no	—	456, 474	442	510	518	0.10	0.02
LC	yes	no	—	337, 421	352, 387^c^	496	511	0.40	(n.d.)^d^

^a^Dark recovery was calculated by fitting data with exponential decay function 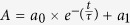, where *t* – time, *A* – absorbance at 474 nm and *a*_*0*_, *a*_*1*_ – scaling coefficients.

^b^Fluorescence spectra and quantum yield measurements were done by excitation at a wavelength of 450 nm for all samples, except for LC where it was 420 nm. Φ_*F*_ - relative quantum yield was calculated with RF as a reference (Φ_*F*_ = 0.27) according to formula 
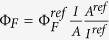
, where *A* – absorbance at excitation wavelength, and *I* – integrated total fluorescence intensity.

^c^LC shows two peak maxima.

^d^Not determined as riboflavin is not a valid reference anymore.
