# Supplementary material for: Structure of a LOV protein in apo-state and implications for construction of LOV-based optical tools
Source: Sci Rep. 2017 Feb 17;7:42971. doi: 10.1038/srep42971 (PMC5314338; doi:10.1038/srep42971)
Supplement: Supplementary Information [file srep42971-s1.doc]

**Supplementary Information**

**Structure of a LOV protein in apo-state and implications for construction of LOV-based optical tools**

Vladimir Arinkin, Joachim Granzin, Katrin Röllen, Ulrich Krauss, Karl-Erich Jaeger, Dieter Willbold, Renu Batra-Safferling

**Affiliations**

**Vladimir Arinkin & Joachim Granzin**

These authors contributed equally to this work

**Institute of Complex Systems, ICS-6: Structural Biochemistry, Forschungszentrum Jülich, 52425 Jülich, Germany**

Vladimir Arinkin, Joachim Granzin, Katrin Röllen, Dieter Willbold & Renu Batra-Safferling

**Institut für Molekulare Enzymtechnologie, Heinrich-Heine-Universität Düsseldorf, Forschungszentrum Jülich, D-52426 Jülich, Germany**

Ulrich Krauss & Karl-Erich Jaeger

**Institut für Bio- und Geowissenschaften, IBG-1: Biotechnologie, Forschungszentrum Jülich, D-52426, Jülich, Germany**

Karl-Erich Jaeger

**Institut für Physikalische Biologie, Heinrich-Heine-Universität Düsseldorf*,* D-40225, Düsseldorf, Germany**

Dieter Willbold

**Present address: Institut für Molekulare Enzymtechnologie, Heinrich-Heine-Universität Düsseldorf, Forschungszentrum Jülich, D-52426 Jülich, Germany**

Katrin Röllen

**Supplementary Figures:**

**Figure S1: Chromophore composition of W619_1-LOV.**


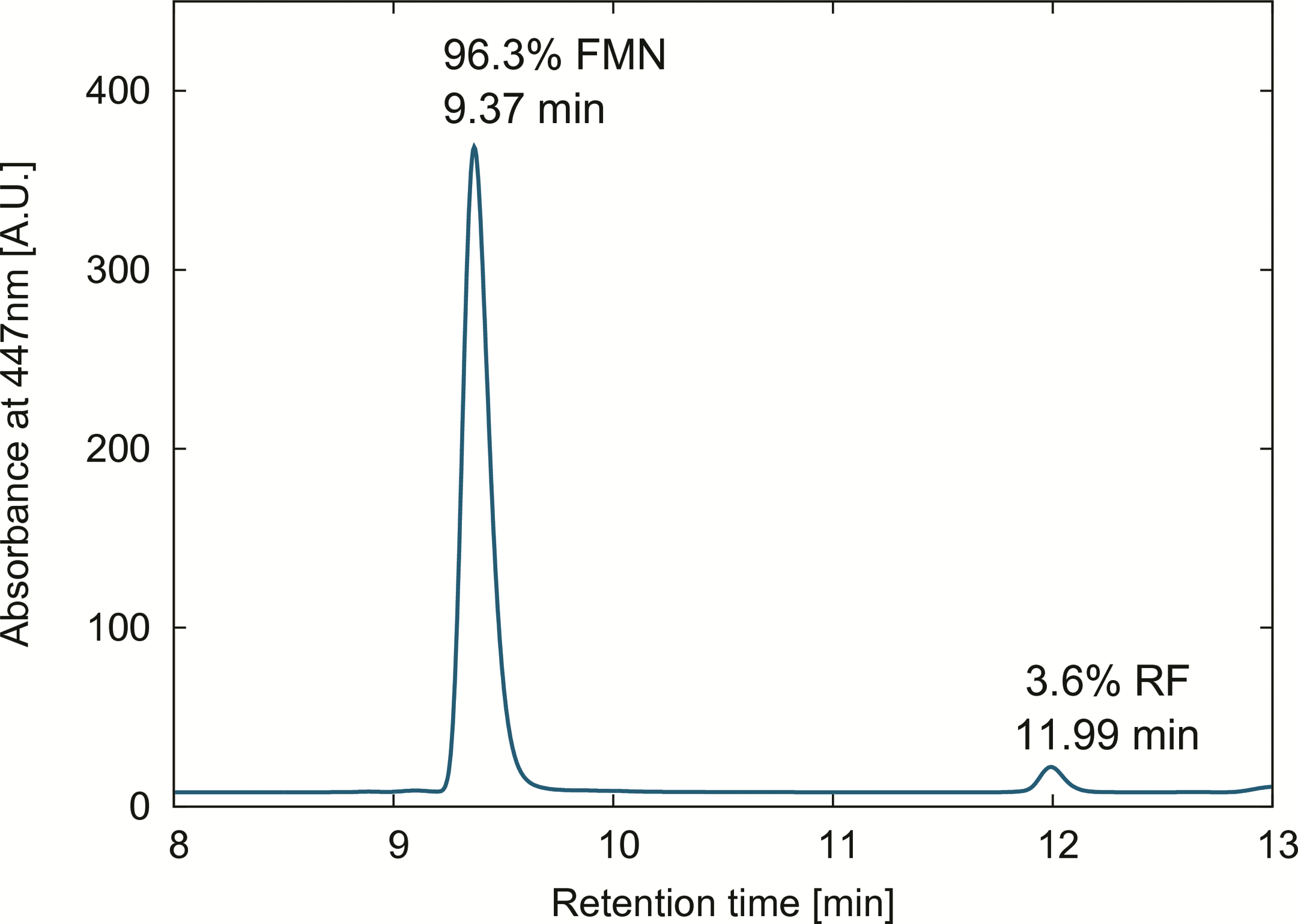


Method: Chromophore composition and quantification of W619_1-LOV was performed by HPLC (Agilent Technologies, Inc., Santa Clara, USA) according to protocol described previously .

Cao Z, Buttani V, Losi A, Gärtner W (2008) A blue light inducible two-component signal transduction system in the plant pathogen Pseudomonas syringae pv. tomato. Biophysical journal 94: 897-905.

**Figure S2: Structures of flavins used as chromophores on W619_1-LOV apo protein.**


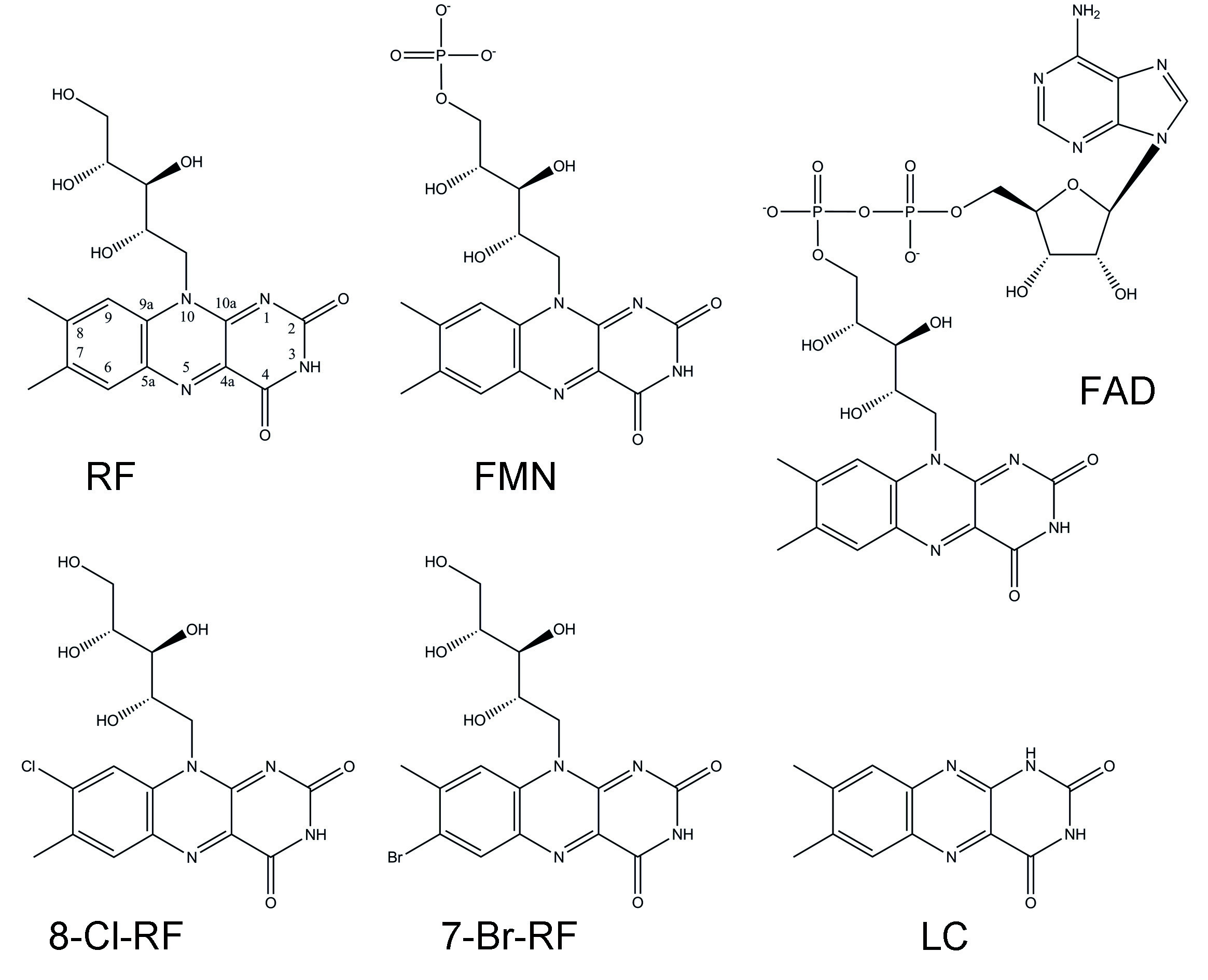


| RF | riboflavin |
| --- | --- |
| FMN | flavin mononucleotide |
| FAD | flavin adenine dinucleotide |
| 8-Cl-RF | 7-methyl-8-chloro-riboflavin |
| 7-Br-RF | 7-bromo-8-methyl-riboflavin |
| LC | lumichrome |

**Supplementary Table:**

**Table S1: X-ray crystal data collection and refinement statistics.**

|  | W619_1-LOV^a^  (5LUV) |
| --- | --- |
| **Data collection** |  |
| Space group | I 4_1_ |
| Cell dimensions   |  |
| *a*, *b*, *c* (Å) | 109.54, 109.54, 95.21 |
|     *α, β, γ* (°) | 90, 90, 90 |
| Resolution (Å) | 47.60 – 2.50 (2.60 – 2.50)^b^ |
| *R*_merge_  *R*_meas_ | 0.101 (1.740)  0.111 (1.908) |
| *I/*σ(*I*) | 12.3 (1.1) |
| *CC*_1/2_ | 0.998 (0.336) |
| Completeness (%) | 99.9 (100.0) |
| Redundancy | 5.7 (5.9) |
|  |  |
| **Refinement** |  |
| Resolution (Å) | 40.56 – 2.50 (2.59 – 2.50) |
| No. reflections | 110498 |
| *R*_work_ / *R*_free_ | 0.21 (0.29) / 0.25 (0.31) |
| No. atoms  (2 monomers per a.u^c^) | 2214 |
| Protein | 2179 |
| Ion | 17 |
| Water | 18 |
| *B* factors (Å^2^) | 76.86 |
| Protein | 76.80 |
| Ion | 100.26 |
| Water | 61.75 |
| R.m.s^d^ deviations |  |
| Bond lengths (Å) | 0.009 |
| Bond angles (°) | 1.02 |

^a^ One crystal was used to collect the complete dataset

^b^ Values in parentheses are for highest-resolution shell

^c^ a.u, asymmetric unit; ^d^ R.m.s, root mean square

**Supplementary Movie:**

**Movie M1**

Structure-based model for conformational differences between the apo state of W619_1-LOV (green) and the dark state of PpSB1-LOV (PDB ID 5J3W, salmon). Mostly, changes can be seen in the secondary structure elements lining the chromophore such as elongation of Fα helix, shortening of the Eα-Fα loop and partial unfolding of the Eα helix. The position of the noncovalently bound FMN chromophore in PpSB1-LOV appears at the end of the movie as stick model and is colored as in Figure 1. This movie is related to Figure 1.

Movie file: W619_1-LOV-to-PpSB1-LOV (MP4 format)
